# The minor collagens in articular cartilage

**DOI:** 10.1007/s13238-017-0377-7

**Published:** 2017-02-17

**Authors:** Yunyun Luo, Dovile Sinkeviciute, Yi He, Morten Karsdal, Yves Henrotin, Ali Mobasheri, Patrik Önnerfjord, Anne Bay-Jensen

**Affiliations:** 1 Biomarkers & Research, Nordic Bioscience A/S, Herlev, Denmark; 2 Faculty of Healthy and Medical Sciences, University of Copenhagen, Copenhagen, Denmark; 3 Department of Clinical Sciences, Medical Faculty, Lund University, Lund, Sweden; 4 Bone and Cartilage Research Unit, Institute of Pathology, Level 5, Arthropole Liège, University of Liège, CHU Sart-Tilman, 4000 Liège, Belgium; 5 Faculty of Health and Medical Sciences, University of Surrey, Guildford, Surrey GU2 7XH UK; 6 Arthritis Research UK Centre for Sport, Exercise and Osteoarthritis, Arthritis Research UK Centre for Musculoskeletal Ageing Research, Queen’s Medical Centre, Nottingham, NG7 2UH UK

**Keywords:** collagen, biomarker, arthritis

## Abstract

Articular cartilage is a connective tissue consisting of a specialized extracellular matrix (ECM) that dominates the bulk of its wet and dry weight. Type II collagen and aggrecan are the main ECM proteins in cartilage. However, little attention has been paid to less abundant molecular components, especially minor collagens, including type IV, VI, IX, X, XI, XII, XIII, and XIV, etc. Although accounting for only a small fraction of the mature matrix, these minor collagens not only play essential structural roles in the mechanical properties, organization, and shape of articular cartilage, but also fulfil specific biological functions. Genetic studies of these minor collagens have revealed that they are associated with multiple connective tissue diseases, especially degenerative joint disease. The progressive destruction of cartilage involves the degradation of matrix constituents including these minor collagens. The generation and release of fragmented molecules could generate novel biochemical markers with the capacity to monitor disease progression, facilitate drug development and add to the existing toolbox for *in vitro* studies, preclinical research and clinical trials.

## ARTICULAR CARTILAGE

Articular cartilage is the most widespread load-bearing cartilage in adults. It is a highly specialized and mechanically resilient connective tissue found on the surface of subchondral bone in diarthrodial joints. Cartilage contains specialized cells called chondrocytes. These cells occupy 1%–3% of the total tissue volume in fully developed tissue and their surrounding extracellular matrix (ECM) is a complex network made up of water, collagen, proteoglycans, and other noncollagenous proteins. Other four types of cartilage are fibroelastic cartilage, fibrocartilage, elastic cartilage, and epiphyseal cartilage.

## COLLAGENS

Collagens are the most abundant family of ECM proteins, which account for two-thirds of the dry mass of adult articular cartilage (Eyre, 2004). Numerous collagen subtypes have been identified in articular cartilage, such as type II, IX, X, XI, VI, XII, and XIV collagen (Van der Rest, 1987). Articular cartilage collagen fibrils mostly consist of type II collagen accompanied with a lesser amount of minor collagens, which provide cartilage with tensile strength and contribute to the physical properties of the mature matrix (Heinegård and Saxne, 2011; Ichimura et al., 2000). However, little is known about the processing of these minor collagens and how their turnover is affected by the progression of osteoarthritis (OA). New knowledge about turnover of those minor collagens will lead to deeper understanding of the dynamics of cartilage turnover, thereby facilitating the development of novel biomarkers that reflect joint health and drug discovery in OA.

In this review, we present an outline of minor collagens in articular cartilage, focusing on the link between these extracellular matrix proteins to OA. Finally, we elaborate on how knowledge of these associations can be used to develop new biomarkers, which provide insight into the translational medicine of OA. Such biomarkers also indicate the effect of a drug on cartilage metabolism and the mode of action. Even though a number of biomarkers already exist, there is a clear medical need for new biomarkers for personalized healthcare (PHC) in OA, biomarkers that more accurately reflect biological activity in different phenotypes of the disease as well as serve as tools in diagnosis and prognosis. This may assist in identification of patients that are in foremost need of treatment and may respond optimally, with the highest efficacy and lowest safety concerns, to a given treatment. Moreover, biomarkers aid pharmaceutical companies develop better targeted therapeutic strategies for selected subpopulations of OA patients. Biomarkers can also enable early decision-making and benchmarking. It is becoming increasingly clear, that one simple marker is insufficient for improved diagnosis, and thus multiple makers that reflect different aspects of the pathophysiology and clinical phenotypes may most likely be needed in combination (Kraus et al., 2015; Karsdal et al., 2014; Henrotin et al., 2016).

**Figure 1:**
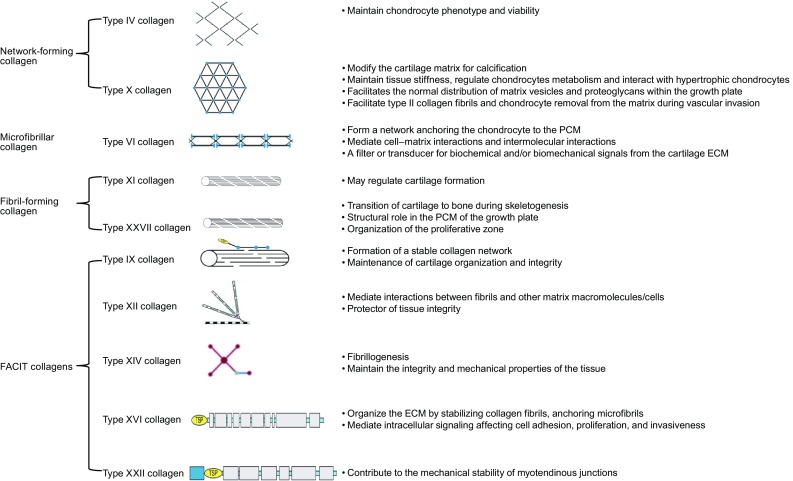
The schematic of minor collagens in articular cartilage. Reproduced with permission from Richard-Blum, S. The collagen family. Cold Spring Harb Perspect Biol 2011;3:a004978. PCM: pericellular matrix; ECM: extracellular matrix

The various types of minor collagens found in articular cartilage are listed in Table 1 and schematically illustrated in Fig. 1.

**Table 1:** Minor collagen overview

Collagen	Distribution in cartilage	Binds to other ECM proteins	Disease-related model	Classifications	Susceptible to Proteinases
Type IV	Pericellular matrix of articular cartilage	Integrins, nidogen, fibronectin, TGF-β (Eyre et al., 2006)	NA	Hexagonal network-forming collagen	MMP-2, 9, 12
Type VI	Pericellular matrix of articular cartilage	Type IV, biglycan, decorin, perlecan, NG2 proteoglycan, fibronectin, tenascin, integrin (Bidanset et al., 1992; Brown et al., 1994)	Col6a1 knockout mice showed accelerated development of osteoarthritis (Wiberg et al., 2001)	Beaded filament collagen (or microfibrillar collagen)	MMP-2, 9
Type IX	Growth-plate cartilage, adult articular cartilage	Matrilin-4, type XII collagen, thrombospondin-4, fibronectin, βig-h3, and epiphycan, type II collagen, COMP, fibronectin, fibromodulin, and osteoadherin (Pfaff et al., 1993; McDevitt et al., 1988; Alexopoulos et al., 2009; Zelenski et al., 2015; Smeriglio et al., 2015; Lee et al., 2013)	Femoral and tibial cartilage in ovalbumin-induced rheumatoid arthritis rabbit model showed significantly reduced type IX collagen content (Wagener et al., 2009) Tibial cartilage in spontaneously osteoarthritic canine had different distribution of type IX collagen compared to healthy tissue (Eyre et al., 1987) Col9a1 knockout mice developed a severe degenerative joint disease resembling human osteoarthritis (Wu et al., 1992)	FACIT	MMP-3, 13
Type X	Hypertrophic zone of the growth plate and basal calcified zone of articular cartilage	Anchorin CII	NA	Hexagonal network- forming collagen	MMP-1, 2, 3, 13
Type XI	Articular cartilage (Eyre et al., 2004; Fässler et al., 1994)	Heparin, heparan sulfate, and dermatan sulfate (Opolka et al., 2007)	Type XI collagen induced mild arthritis in DBA/1 mice (Hagg et al., 1997) Immunizing type XI collagen induced chronic arthritis, IgG deposits in cartilage, and joint destruction in the Lewis rat (Czarny-Ratajczak et al., 2001)	Fibril-forming collagen	MMP-2
Type XII		Decorin, fibromodulin, tenascin-X, COMP (Lohiniva et al., 2000; Kuivaniemi et al., 1997)	NA	FACIT	NA
Type XIV	Uniformly throughout the articular cartilage (Nakata et al., 1993)	Decorin and type I collagen (Kojima et al., 2001)	NA	FACIT	MMP-13
Type XVI	Territorial matrix of chondrocytes (Poole et al., 1997)	Types II and XI collagen, fibrillin-1 and fibronectin (Poole et al., 1997; Mustafa et al., 2000)	NA	FACIT	NA
Type XXII	Articular surface of joint cartilage	Fibrillins, integrins (α2β1 and α11β1) (Loughlin et al., 2002; Alizadeh et al., 2005)	NA	FACIT	NA
Type XXVII	Proliferative zone chondrocytes (Boissier et al., 1990)	NA	Knockdown of COL27a1 in zebrafish embryos delayed and decreased vertebral mineralization, morphological abnormalities and scoliosis (Yamagata et al., 1991)	Fibril-forming collagen	NA

FACIT = Fibril-associated collagens with interrupted triple helices. COMP = cartilage oligomeric matrix protein. NA = Not available

## DISTRIBUTION, STRUCTURE, AND FUNCTION OF MINOR COLLAGENS

### Type VI collagen—a microfibrillar collagen

Although it only makes up 1% of total collagen in adult articular cartilage (Eyre et al., 2006), type VI collagen is mainly enriched in the pericellular matrix (PCM), involving the attachment and integrity of chondrocytes. Type VI collagen is able to bind to a wide variety of ECM proteins, including type II collagen (Bidanset et al., 1992), type XIV collagen (Brown et al., 1994), matrilin-1 (Wiberg et al., 2001), and decorin (Bidanset et al., 1992), thereby forming a network that anchors the chondrocyte to the PCM in articular cartilage. Due to the high affinity with numerous ECM components and cell membrane (Bidanset et al., 1992; Wiberg et al., 2001), type VI collagen has been hypothesized to play important roles in mediating cell–matrix interactions and intermolecular interactions (Pfaff et al., 1993).

The precise role of collagen VI has not yet been clearly defined. However, type VI collagen may serve as a filter or transducer for biochemical and/or biomechanical signals from the cartilage ECM. Type VI collagen with lower molecular weight was evident in the pathological osteoarthritic dogs sacrificed 3, 5, and 7 months after surgery in comparison to the controls (McDevitt et al., 1988). It indicated that degradation products of larger type VI chains might be significant in the role this molecule plays in osteoarthritis. The type VI collagen-deficient mice (*Col6a1*) exhibited accelerated development of hip osteoarthritis, a delayed secondary ossification process, and a loss of the stiffness of the articular cartilage PCM (Alexopoulos et al., 2009). Type VI collagen demonstrated an important role in regulating the physiology of the synovial joint. In another context, the deficiency of type VI collagen in mice resulted in decreased stiffness and increased chondrocyte swelling (Zelenski et al., 2015). These findings suggest that type VI collagen has essential roles in transmitting mechanical and osmotic stresses from the ECM to the chondrocytes (Zelenski et al., 2015). The soluble type VI collagen was reported to promote chondrocyte proliferation under both healthy and osteoarthritic conditions. However, proliferation was not observed upon treatment of immobilized type VI collagen in chondrocytes, indicating that soluble type VI collagen can be applied for autologous chondrocyte implantation to expand chondrocytes (Smeriglio et al., 2015). It was reported that a variant in the human *Col6a4* gene is associated with knee OA in Japanese and Chinese populations, but not found in a Korean population (Lee et al., 2013) nor in European OA individuals (Wagener et al., 2009). The contradictory findings could be explained by either the ethnic differences in OA susceptibility genes or the differences of criteria in OA selection.

### Type IX, XII, XIV, XVI, and XXII collagen—the FACIT collagens

These collagens are members of the fibril-associated collagen with interrupted triple helix (FACIT), which do not form fibrils by themselves, but are associated with the surface of various fibrils.

#### Type IX collagen

Type IX collagen simply makes up 1%–5% of total collagen in adult articular cartilage and 10% of that in fetal cartilage (Eyre et al., 2006). It is usually found in tissues containing type II collagen, like growth plate cartilage and adult articular cartilage (Eyre et al., 1987). It forms the unique hetero fibril network in the matrix of cartilage via association with type II and type IX collagen (Wu et al., 1992). Type IX collagen is extensively cross-linked with type II collagen through the lysyl oxidase mechanism (Wu et al., 1992). Eyre et al. discovered that the covalent cross-linking formed at the N-telopeptide of α1(II) chain and the COL1 in all three chains of type IX collagen in both human and bovine cartilage (Eyre et al., 2004). Additionally, Eyre et al. also observed the binding inter type IX collagen molecules at the COL2 domain and the non-collagenous globular domain (NC1) domain (Wu et al., 1992).

Mice with a completely inactivated *Col9a1* gene showed no detectable abnormalities at birth but thereafter had a severe degenerative joint disease resembling human OA at 4-months or older (Fässler et al., 1994). In a different context, the knockout of type IX collagen altered the time course of callus differentiation during bone fracture healing, and delayed the maturation of cartilage matrix (Opolka et al., 2007). A deficiency of α1(IX) in mice has been shown to lead to instability of hyaline cartilage (Hagg et al., 1997). Other studies suggested that mutations of α1(IX) (Czarny-Ratajczak et al., 2001), α2(IX), and α3(IX) (Lohiniva et al., 2000) can elicit multiple epiphyseal dysplasia, a heterogeneous skeletal disorder with early-onset OA as a manifestation. Mutations in the Col9a2 are also linked to multiple epiphyseal dysplasia characterized by symptoms ranging from pain and stiffness in joints to OA (Kuivaniemi et al., 1997). In another experiment, the mutated type IX collagen had a mild chondrodysplasia (Nakata et al., 1993). In a rabbit model of ovalbumin-induced rheumatoid arthritis, the NC4 domain of type IX collagen content was reduced in femoral and tibial cartilage, revealing early damage of type IX collagen in articular cartilage following induction of joint inflammation (Kojima et al., 2001). The immunohistochemical staining of type IX collagen in normal mature and spontaneously osteoarthritic canine tibial cartilage revealed that changes in type IX collagen distribution played crucial role in the chondron remodeling and chondrocyte cluster formation associated with osteoarthritic degeneration (Poole et al., 1997). All these findings highlight that type IX collagen may play important roles in the pathogenesis of arthritis diseases, the formation of a stable collagen network and in the maintenance of cartilage organization and integrity. In humans, Col9a1 has been identified as a susceptibility locus for female hip OA (Mustafa et al., 2000; Loughlin et al., 2002; Alizadeh et al., 2005), suggesting that Col9a1 is involved in hip OA. The decreased expression of type IX collagen in the cartilage may render the matrix more subject to mechanical forces, thereby resulting in the pathogenesis of human OA. Type IX collagen is also used to induce chronic arthritis in the DBA/1 mice (Boissier et al., 1990).

Taken together, type IX collagen is crucial for the maintenance of cartilage matrix and formation of collagen meshwork. Turnover of type IX collagen by proteases is an early event in degenerative joint disease. The reduced level of type IX collagen may contribute to the pathogenesis of OA.

#### Type XII collagen

Type XII collagen shares structural homologies with type IX and type XIV collagens (Yamagata et al., 1991). Additionally, in common with several other FACITs, the length of α1(XII) chains is affected by complex alternative splicing of type XII collagen primary transcripts. As a result, two distinct forms of type XII collagen—short(XIIB) and long(XIIA)—are generated. Although both forms of type XII collagen are present in cultured fibroblasts—the expression of long or short form being determined by whether cells grow in monolayer or 3D culture—the long transcript variant predominates. The long form is also the only type XII collagen variant expressed in human fetal chondrocytes (Keene et al., 1991).

In terms of biological function, type XII collagen has been implicated in fibril formation, cell adhesion, fibrosis and osteogenesis, and in areas of high mechanical stress may serve as a protector of tissue integrity (Chiquet et al., 2014; Arai et al., 2008). Immunohistochemistry staining and fibrillogenesis studies show that type XII collagen can be incorporated into type I collagen fibrils in dense connective tissues and bone. It potentially helps to mediate interactions between fibrils and other matrix macromolecules/cells, or acts as a ‘shock-absorber’ similar to proteoglycans in cartilage (Arai et al., 2008; Taylor et al., 2014). Type XII collagen associates with articular cartilage and growth plate region during rat forelimb development, and may be necessary for microenvironment that supports hyaline cartilage formation (Taylor et al., 2014; Gregory et al., 2001). Type XII collagen also has been shown to be present in the secretome of human passaged chondrocytes (Polacek et al., 2011), however, not in the secretome of cartilage explants (Taylor et al., 2014).

#### Type XIV collagen

Type XIV collagen is a large, non-fibrillar ECM protein, structurally similar to type XII collagen. In cartilage, a population of type XIV collagen exists a chondroitin sulfate proteoglycan, since it was sensitive to chondroitinases ABC and AC treatments (Watt et al., 1992).

Type XIV collagen is prevalent within connective tissues that contain large amounts of fibrillar collagens, where it localizes near the surface of banded collagen fibrils (Nishiyama et al., 1994). Immunofluorescence localization showed that type XIV collagen was prominent at the ligament-bone junction, and in bovine cartilage. Type XIV collagen localizes relatively uniformly throughout the articular cartilage, but is absent from growth plate regions (Watt et al., 1992).

In addition to reported interactions with type I, II, V, and VI collagens, type XIV collagen also interacts with heparin, CD44, and cartilage oligomeric matrix protein (COMP) (Giry-Lozinguez et al., 1998). Type XIV collagen is predominantly expressed in differentiated tissues and late embryonic development. Ruehl et al. postulated that it is involved in tissue differentiation, and particularly, its first FN-III domain are potent inducers of reversible cellular quiescence and differentiation in human and mouse mesenchymal cells (Ruehl et al., 2005). Their study saw reduction of *de novo* DNA synthesis without alterations to cell numbers and viability and restoration of maximal proliferation upon serum supplementation. Similarly to type XII collagen, type XIV collagen is often found in areas of high mechanical stress (Hemmavanh et al., 2013), and has roles in fibrillogenesis and maintaining the integrity and mechanical properties of the tissue (Chen et al., 2015).

#### Type XVI and XXII collagen

Type XVI collagen has been identified in the territorial matrix of the chondrocytes, associating with thin weakly banded collagen fibrils containing types II and XI collagen (Kassner et al., 2003). In cartilage, type XVI collagen is a component of small heterotypic D-banded fibrils (Kassner et al., 2003) and is strongly expressed in differentiating chondrocytes (Lai and Chu, 1996). Type XVI collagen may be incorporated into structurally and functionally discrete matrix aggregates in cartilage. Its main function is to organize the ECM by stabilizing collagen fibrils, anchoring microfibrils, mediating intracellular signalling affecting cell adhesion, proliferation, invasiveness as well as the formation of focal adhesions.

It has been shown that N-terminal processing of type XVI collagen results in 182 kDa and 78 kDa fragments (Kassner et al., 2004), whereas C-terminal and subsequent N-terminal processing results in 150 kDa, 110 kDa, 50 kDa, and 35 kDa fragments, respectively (Grässel et al., 1996). Such fragments may be utilized as targets of biochemical markers for cartilage biology.

Type XXII collagen is expressed at the junction between synovial fluid and surface of articular cartilage (Koch et al., 2004) and associated with the extrafibrillar matrix in cartilage. Type XXII collagen is also detectable in human arthritic joints, but the immunofluorescence staining pattern is broadened and fuzzy. Unlike other FACIT collagens, type XXII collagen interacts with microfibrils, such as fibrillins or type VI collagens instead of collagen fibrils (Koch et al., 2004). Its function remains unknown, but may contribute to the mechanical stability of myotendinous junctions (Koch et al., 2004; Zwolanek et al., 2014). Type XXII collagen could serve as a marker to explore pathologic processes of joint diseases and to study tissue junction formation during development and regeneration of cartilage due to its expression location.

### Type IV and X collagen—network-forming collagen

Type IV collagen is a network forming collagen, which is exclusively found in the pericellular matrix of normal articular cartilage, and osteoarthritic articular cartilage in human and goat (Foldager et al., 2014; Jeng et al., 2013; Kvist et al., 2008). However, controversially it was reported to be absent in any human cartilage subtypes including hyaline, fibrous, and elastic cartilage (Wachsmuth et al., 2006). Overexpression of regulator of MMP-13 increased the expression of type IV collagen in chondrocytes (Wang et al., 2015). Type IV collagen may be involved in maintaining chondrocyte phenotype and viability and provide clues to the progression of degenerative joint disorders (Kvist et al., 2008).

Fragments originating from type IV collagen released by protein remodeling have been thoroughly investigated for their uses as biomarkers. Several formation and degradation biomarkers, e.g. C4M, C4M2 (Karsdal et al., 2015), C4M3a (Sand et al., 2016), C4M12a1, C4M12a3 (Sand et al., 2013), P4NP 7S (Leeming et al., 2013), and Tumstatin (Hamano et al., 2003) have been developed, indicating the role of type IV collagen turnover in most connective tissue diseases.

Type X collagen is a homotrimeric collagen, which consists of three identical α1(X) chains with 3 domains each, a short triple helix, an NC1 at C-terminus, and a short non-helical at N-terminus (NC2) (Shen, 2005). These structures are believed to play a key role in modifying the cartilage matrix for the subsequent bone formation during endochondral ossification (Kwan et al., 1991). Type X collagen is inclined to be cleaved by interstitial collagenase, gelatinase, human neutrophil elastase, pepsin, and trypsin (Frischholz et al., 1998).

Type X collagen constitutes about 1% of total collagen in adult articular cartilage (Eyre, 1991). It is revealed that 45% of the total collagens produced by mature hypertrophic chondrocytes are type X collagen (Shen, 2005). As a specific collagen in cartilage, type X collagen is synthesized by hypertrophic chondrocytes and is found exclusively in the hypertrophic cartilage and the calcified zone of articular cartilage (Gannon et al., 1991). Increased expression has been observed in arthritis as the chondrocytes become hypertrophic (Shen, 2005; Kwan et al., 1991; Frischholz et al., 1998; Eyre, 1991; Gannon et al., 1991; van der Kraan and van den Berg, 2012). Hypertrophic chondrocytes express a variety of proteins and enzymes including type X collagen, matrix metalloproteinase 13, alkaline phosphatase, which do not seem to exist in normal proliferating chondrocytes (Steinert et al., 2009; D’Angelo et al., 2000). As the most widely used marker for chondrocyte hypertrophy (Alvarez et al., 2000), type X collagen is normally expressed in human OA cartilage especially in the vicinity of lesions, but not in human healthy articular cartilage (Brew et al., 2010). Interestingly, Fukui et al. observed that the expression of *Col10a1* was lower in the more degenerated OA cartilage than in the less degenerated area (Fukui et al., 2008, 2008). The expression of type X collagen has been reported to be upregulated in experimental animal OA models (Matsumoto et al., 2009; Huebner et al., 2009) and human OA cartilage as well (Walker et al., 1995). However, other studies have shown that the expression of type X collagen in late stage osteoarthritic cartilage was not significantly elevated in human and rat OA (Brew et al., 2010; Appleton et al., 2007). A possible explanation for this discrepancy could be that chondrocyte hypertrophy-like change possibly only exists in a subset of human OA patients.

The biological function of type X collagen is thought to maintain tissue stiffness, regulate chondrocytes metabolism and interact with hypertrophic chondrocytes (Luckman et al., 2003). It also facilitates the process of calcification, the normal distribution of matrix vesicles and proteoglycans within the growth plate. Mutations in the type X collagen have been found in patients with Schmid metaphyseal chondrodysplasia, an autosomal dominant cartilage disorder with symptoms of coxa vara, short stature, and a waddling gait (Kuivaniemi et al., 1997). Given its restricted localization in the hypertrophic zone of the growth plate, type X collagen appears to support endochondral bone growth and development during the degradation of ECM in cartilage. It also participates in the matrix calcification, facilitate type II collagen fibrils removal and chondrocyte removal from the matrix during vascular invasion (Schmid and Linsenmayer, 1985).

### Type XI and XXVII collagen—the fibril-forming collagen

Type XI collagen is primarily cross-linked to each other in cartilage. The cross-linkages result in the formation of mature type XI collagen fibers with the help of type II and IX collagen. It is broadly distributed in articular cartilage, tendons, trabecular bone, and skeletal muscle (Mio et al., 2007). Like the other fibril-forming collagens, type XI collagen is synthesized as a procollagen which is subsequently degraded to the mature form depositing into the ECM (Sussman et al., 1984). The absence in the α chain of type XI collagen leads to abnormally thickened cartilage fibril (Hida et al., 2014) and OA (Rodriguez-Fontenla et al., 2014; Jakkula et al., 2005). It has been shown that a type XI collagen mutation results in increased degradation of type II collagen in articular cartilage (Lu et al., 2002).

Type XI collagen accounts for 3% to 10% of total collagen in adult articular cartilage and fetal cartilage, respectively (Eyre, 2002). It is preferentially retained at the chondrocyte surface and involved in the organization of the pericellular matrix via interaction with cartilage proteoglycans (Smith et al., 1989). In embryonic cartilage, type XI collagen has a uniform diameter of ~20 nm and diameter control is regulated by the proportion of collagen II and XI while collagen IX strongly increase the efficiency of fibril formation (Blaschke et al., 2000). The thin fibrils in embryonic cartilage are constructed from a 10 + 4 microfibrillar arrangement (central core of 2 microfibrils each of type II and type XI collagen) (Holmes and Kadler, 2006). This arrangement explains why the narrow fibrils are lacking in collagen XI knockout animals.

The mutation of type XI collagen in mice leads to Stickler’s syndrome, an autosomal dominant disorder with symptoms of mild spondyloepiphyseal dysplasia, OA, and sensorineural hearing loss (Kuivaniemi et al., 1997). In other experiments, the mice lacking type XI collagen exhibited age-dependent OA-like changes in knee and temporomandibular joints of heterozygous cho/+ mice (Xu et al., 2003, 2005). Mutations in *Col11a1* and *Col11a2* have also been shown to result in relatively mild chondrodysplasias associated with OA (Myllyharju and Kivirikko, 2001). In addition, two single-nucleotide polymorphisms (SNPs) in *Col11a1* showed significant association with hip OA in a meta-analysis of nine genome-wide association studies (Rodriguez-Fontenla et al., 2014). Type XI collagen is often used to induce chronic arthritis in the DBA/1 mouse and rat (Cremer et al., 1994). Interestingly, type XI collagen was shown to be arthritogenic in Adderley Park rats but not in Sprague-Dawley rats, although type II collagen-induced arthritis in both strains (Morgan et al., 1983). Lu et al. observed that immunization of rats with homologous type XI collagen led to chronic and relapsing arthritis with different genetics and joint pathology than arthritis induced with homologous type II collagen (Lu et al., 2002).

Although the role of type XI collagen in the formation of cartilage collagen fibrils remains unclear, type XI collagen may regulate cartilage formation in that it is the first cartilage collagen deposited by mesenchymal stem cells undergoing chondrogenic differentiation (Xu et al., 2008).

Type XXVII collagen is prominently located at sites of transition from cartilage to bone (Pace et al., 2003; Boot-Handford et al., 2003) and in the matrix surrounding proliferative chondrocytes in the epiphyseal growth plate (Plumb et al., 2011). The expression of type XXVII collagen is regulated by factors SOX9 and Lc-Maf in chondrocytes (Mayo et al., 2009; Jenkins et al., 2005).

In developing endochondral bone, type XXVII collagen plays a role in the transition of cartilage to bone during skeletogenesis (Hjorten et al., 2007). It is also believed to play a key structural role in the pericellular extracellular matrix of the growth plate and is required for the organization of the proliferative zone (Plumb et al., 2011).

## MINOR COLLAGEN METABOLITES AS BIOCHEMICAL MARKERS OF JOINT DISEASE

Extracellular matrix remolding (ECMR) is a delicate equilibrium and a prerequisite for maintenance of a healthy tissue, in which old proteins continuously are degraded and new proteins are formed (Karsdal et al., 2013). This delicate balance may be disturbed in connective tissues disease, resulting in an altered turnover of both formation and degradation, leading to a tissue imbalance. Irreversible degradation in the cartilage collagen network is believed to be a critical event involved in the pathophysiological progress of arthritis. During tissue remodeling, proteases release small protein fragments into the circulation that may be used as serological biomarkers of tissue degradation (Karsdal et al., 2013). A sub-set of pathological proteases are over-expressed in the affected tissue area, resulting in release of protease specific fragments of signature proteins of the arthritis ECM (Karsdal et al., 2010). These fragments may be utilized as early diagnostic or prognostic serological markers, as they originate from the structure of cartilage, which in part is the consequence of disease.

Although accounting for only a small fraction of the mature matrix, minor collagens not only play structural roles in the mechanical properties, organization, and shape of articular cartilage, but also have specific biological functions. Genetic studies of these minor collagens in articular cartilage reveal they are associated with degenerative joint disease. The progressive destruction of cartilage involves the degradation of matrix constituents including these minor collagens. We speculate that the release of fragmented molecules from minor collagen could be potential complementary biomarkers of the existing one. It has been shown that pro-peptides of type VI collagen are released during collagen synthesis (Sun et al., 2015; Sand et al., 2015). However, whether pro-peptides of other minor collagens exist is still unknown. Many minor collagens of articular cartilage have been shown to be susceptible to degradation by MMPs (Eckhard et al., 2016), e.g. IV (Karsdal et al., 2013), VI, IX, and X collagen (He et al., 2014; Schmid et al., 1986). The degradation products of type IX collagen have been investigated in *in vitro*, *ex vivo*, and *in vivo* cartilage models. An MMP-3 cleavage site within NC2 domain was revealed *in vitro* (Wu et al., 1991). D. Heinegård and colleagues observed two MMP-13 cleavage sites within NC4 and COL3 domain respectively, in a bovine nasal cartilage *ex vivo* induced by interleukin-1 (IL-1) (Danfelter et al., 2007). They claimed that these degradation events precede the major loss of type II collagen. This cleavage, which released NC-4 fragments into synovial fluid and serum of patients with OA or rheumatic arthritis (RA), caused the collagen network swelling seen in articular cartilage in early experimental OA. Type X collagen is subject to interstitial collagenase and gelatinase cleavage at two distinct sites within triple helix domain (Goldring et al., 2013). He et al. reported that C-Col 10, which is a C-terminal fragment of the NC1 domain in type X collagen, significantly elevated in OA patients compared to healthy subjects (He et al., 2014; Gudmann et al., 2016). Type XI collagen is resistant to collagenase but hydrolysed by gelatinases resulting in a number of degradation products. These events were believed to play a vital role in the turnover of articular cartilage in health and disease states. Type VI collagen was reported to be susceptible to degradation by MMP2 and MMP9 (Veidal et al., 2011).

The collagen of articular cartilage is a co-polymeric network of different types of collagen that interact specifically at the molecular level. Types II, IX, and XI collagen are cross-linked together, forming the extracellular framework of the tissue. Cross-linking plays an important role in the ECM meshwork, especially for the fibrillar collagens (types I–III) and minor collagens (types IV–XIV), and thereby in tissue integrity. Type XII and XIV collagen can be extracted without proteolysis, so they appear not to be covalently polymerized in the matrix (Watt et al., 1992), but are thought to bind physically to collagen fibril surfaces via their COL1/NC1 domains. It is vital for collagen to be able to cross-link with the neighboring collagen and/or other ECM components (Reiser et al., 1992). Understanding the details of ECM remodeling mechanisms in cartilage is critical for knowing the pathological process of joint diseases. ECMR is a continuous and dynamic process of cartilage development, maintenance, and pathogenesis. It results in uniquely modified proteins during the pathogenesis of disease. Specific proteolytic activities are required for a range of cellular functions and interactions with the ECM. However, in pathological condition, proteolysis of collagen framework is integral to the process of cartilage destruction and joint failure. So in theory, a range of type II, IX, and XI collagen metabolites could be exploited as molecular biochemical markers in arthritis.

## PERSPECTIVES

Our understanding of the biology of joint disease has been hampered by the lack of well-characterised biomarkers that perform well in clinical studies. Imaging markers, e.g. radiographs, which are the traditional method of defining clinical arthritis, can only detect advanced, relatively gross changes in joint anatomy and joint space narrowing only after significant deterioration has already taken place. According to the FDA critical path, there is an unmet need for the development of novel diagnostic and prognostic OA biomarkers for use in clinical trials (Karsdal et al., 2009). A strong prognostic or burden of disease biomarker for osteoarthritis would be of great value to healthcare all over the world, as the prevalence of OA is continuously increasing.

Therefore biochemical markers are receiving increased attention for their capability to detect earlier stages of the disease process, monitor the progress of destruction and prognose the development of arthritis, accurately and relatively quickly assess the efficacy of therapy. Recently the US National Institutes of Health (NIH)-industry partnership funded by the OA Biochemical Markers Network (Bauer et al., 2006; van Spil et al., 2010) proposed the BIPED (Burden of disease, Investigative, Prognostic, Efficacy of intervention, and Diagnostic) classification system. It seems unlikely that any single marker can offer sufficient sensitivity and specificity to predict the progression of arthritis and detect response to medical treatment. This classification system will help to improve the capability to develop and analyze arthritis biomarkers (Henrotin et al., 2016; Bay-Jensen et al., 2016; Kraus et al., 2015).

The release of protease degradation products provides exciting opportunities for monitoring disease progression in arthritis patients, and to investigate whether these fragments are involved in facilitating the existing pathology, for example, by inducing inflammation. As the fragmented molecules of type II collagen have shown promise as molecular markers of joint disease, it is likely that identification of cleavage fragments and other post-translational modifications (PTMs), including cross-linking and isomerization from various minor collagens in cartilage may produce unique joint disease-specific biomarkers.

Development of simple and reliable non-invasive biomarkers of OA is an important goal in clinical rheumatology and will facilitate the design and evaluation of clinical trials on disease modifying osteoarthritis drugs (DMOADs). Biomarkers that measure the stages and phenotypes of OA and, ideally, predict risk of joint-related outcomes would significantly improve decision-making in terms of dosing, treatment time, risk/benefit ratio, and transfer knowledge to label. By implementing biochemical markers in all stages of drug development, novel drug candidates may be identified at early decision points and potential safety issues may be addressed in a timely way, thereby increasing efficiency, reducing costs and prompting efficient allocation of limited resources. Thus, there is a need for different types of biochemical markers for different stages of drug development in OA.

In conclusion, development of biomarkers assessing the turnover of minor collagens may provide novel and translational diagnostic tools for investigating the effect of known drug targets on cartilage in preclinical or clinical settings, thereby providing proof of principle for test of those drugs in OA clinical trials.
